# Fatty acid transport protein 2 interacts with ceramide synthase 2 to promote ceramide synthesis

**DOI:** 10.1016/j.jbc.2022.101735

**Published:** 2022-02-16

**Authors:** Jiyoon L. Kim, Beatriz Mestre, Sergey Malitsky, Maxim Itkin, Meital Kupervaser, Anthony H. Futerman

**Affiliations:** 1 Department Biomolecular Sciences, Weizmann Institute of Science, Rehovot, Israel; 2 Life Sciences Core Facilities, Weizmann Institute of Science, Rehovot, Israel; 3 Nancy and Stephen Grand Israel National Center for Personalized Medicine, Weizmann Institute of Science, Rehovot, Israel

**Keywords:** ceramide, ceramide synthase, dihydroceramide, fatty acid transport protein, sphingolipids, CerS, ceramide synthase, ER, endoplasmic reticulum, FATP, fatty acid transport protein, FB1, fumonisin B1, Hek, human embryonic kidney, LC, long-chain, SL, sphingolipid, VLC, very long-chain

## Abstract

Dihydroceramide is a lipid molecule generated *via* the action of (dihydro)ceramide synthases (CerSs), which use two substrates, namely sphinganine and fatty acyl-CoAs. Sphinganine is generated *via* the sequential activity of two integral membrane proteins located in the endoplasmic reticulum. Less is known about the source of the fatty acyl-CoAs, although a number of cytosolic proteins in the pathways of acyl-CoA generation modulate ceramide synthesis *via* direct or indirect interaction with the CerSs. In this study, we demonstrate, by proteomic analysis of immunoprecipitated proteins, that fatty acid transporter protein 2 (FATP2) (also known as very long-chain acyl-CoA synthetase) directly interacts with CerS2 in mouse liver. Studies in cultured cells demonstrated that other members of the FATP family can also interact with CerS2, with the interaction dependent on both proteins being catalytically active. In addition, transfection of cells with FATP1, FATP2, or FATP4 increased ceramide levels although only FATP2 and 4 increased dihydroceramide levels, consistent with their known intracellular locations. Finally, we show that lipofermata, an FATP2 inhibitor which is believed to directly impact tumor cell growth *via* modulation of FATP2, decreased *de novo* dihydroceramide synthesis, suggesting that some of the proposed therapeutic effects of lipofermata may be mediated *via* (dihydro)ceramide rather than directly *via* acyl-CoA generation. In summary, our study reinforces the idea that manipulating the pathway of fatty acyl-CoA generation will impact a wide variety of down-stream lipids, not least the sphingolipids, which utilize two acyl-CoA moieties in the initial steps of their synthesis.

The sphingolipid (SL) biosynthetic pathway begins with the condensation of an amino acid, normally serine, with a fatty acyl-CoA, normally palmitoyl CoA, to generate 3-ketosphinganine, followed by the action of 3-ketosphinganine reductase to generate sphinganine (1). The latter is then *N*-acylated by (dihydro)ceramide synthases (CerSs), which can also *N*-acylate the sphingosine that is generated in the recycling pathway (2). Thus, dihydroceramide generation requires the input of two acyl-CoAs. Whereas serine palmitoyl transferase has a relativity-restricted ability to use acyl-CoAs of different chain lengths (3), the CerS can use a much broader variety of acyl-CoAs (4). For instance, of the six mammalian CerS (5), CerS2 uses very long-chain (VLC) acyl-CoAs (*i.e.*, C22–26) (6), whereas CerS5 and 6 use long-chain (LC) acyl-CoAs (*i.e.*, C16) (7, 8).

Unexpected and complex modes of regulation of this pathway have been uncovered over the past few years. Thus, CerS can be regulated *via* dimerization (9) or phosphorylation (10), or *via* interactions with other proteins, including a number related to either the elongation of acyl-CoAs, such as ELOVL1 (11), the supply of acyl-CoAs, such as acyl-CoA–binding protein (12), and generation of LC-acyl-CoAs *via* ACSL5 and DGAT2, which are subsequently incorporated into 1-*O*-acyl-ceramides (13).

We now identify an additional protein family which directly interacts with the CerSs and specifically with CerS2, namely VLC–acyl-CoA synthetase, encoded by *Slc27a2*, also known as fatty acid transport protein 2 (FATP2). FATP2 is one of the six members of the FATP family (14), bifunctional proteins that mediate the ATP-dependent import of LC-fatty acids into the cell (15, 16) as well as catalyzing the conversion of LC- and VLC-fatty acids to acyl-CoA thioesters (17). FATPs can be distinguished from LC-acyl-CoA synthetases by their VLC–fatty acyl-CoA synthetase activity (17, 18, 19). Furthermore, members of the FATP family differ in their tissue distribution, levels of expression in different cell types, and in subcellular localization (20).

We now demonstrate that the FATPs directly interact with the CerS and mainly focus on the interaction between FATP2 and CerS2, which depends on the catalytic activity of both. Moreover, inhibition of FATP2 using lipofermata decreases ceramide synthesis, suggesting that some of the effects ascribed to FATP2 inhibition in various cancers (21, 22) may actually be mediated *via* the ceramide pathway.

## Results

### FATPs interact with CerS2 and modulate cellular SL levels

To identify proteins that may interact with CerS2, we analyzed proteins that were immunoprecipitated from mouse liver homogenates using an anti-CerS2 antibody. Three hundred seventy-nine proteins were identified by LC-MS/MS (Table S1). Comparison with proteins immunoprecipitated from homogenates obtained from CerS2 null mouse liver (23) revealed 15 proteins that were differentially immunoprecipitated >1.5-fold (Table 1). Among these were CerS2, as expected, and a number of proteins which do not seemingly appear to be related to the SL or associated metabolic pathways, such as glutamine synthetase and retinal dehydrogenase. These latter proteins may either interact nonspecifically with CerS2 upon immunoprecipitation after homogenization in 1% digitonin or may be associated with the CerS *via* as yet unknown mechanisms. However, a number of the immunoprecipitated proteins appeared to be *bone fide* candidates to interact with the SL metabolic pathway, including FATP2. Since acyl-CoAs are one of the two substrates used in the generation of dihydroceramide by CerS2 (2), the 6.6-fold ratio of immunoprecipitation (Table 1) of FATP2 in WT *versus* CerS2 null mouse liver is supportive of a relationship and possible direct interaction between FATP2 and CerS2.

**Table 1:** Putative CerS2-interacting proteins identified by LC-MS/MS

Protein ID	Protein	Gene	Ratio WT:CerS2 null	*p* value
Q924Z4	Ceramide synthase 2	CerS2	227	<0.001
P15105	Glutamine synthetase	Glul	103	<0.01
Q91XD4	Formimidoyltransferase-cyclodeaminase	Ftcd	8.52	<0.05
O35488	Very long-chain acyl-CoA synthetase	Slc27a2	6.58	<0.05
P24549	Retinal dehydrogenase 1	Aldh1a1	5.22	<0.01
P16015	Carbonic anhydrase 3	Ca3	4.13	<0.01
P33267	Cytochrome P450 2F2	Cyp2f2	3.68	<0.05
P17563/Q63836^a^	Selenium-binding protein 1/Selenium-binding protein 2	Selenbp1/Selenbp2	3.07	<0.01
O35490	Betaine–homocysteine S-methyltransferase 1	Bhmt	2.88	<0.05
O08709	Peroxiredoxin-6	Prdx6	2.49	<0.05
P00329	Alcohol dehydrogenase 1	Adh1	2.25	<0.05
Q9WVL0	Maleylacetoacetate isomerase	Gstz1	2.24	<0.05
P54869	Hydroxymethylglutaryl-CoA synthase, mitochondrial	Hmgcs2	2.15	<0.005
P17751	Triosephosphate isomerase	Tpi1	2.15	<0.01
Q8BWT1	3-ketoacyl-CoA thiolase, mitochondrial	Acaa2	1.72	<0.05

Proteins were immunoprecipitated from WT mouse liver homogenates using an anti-CerS2 antibody and compared to those immunoprecipitated from CerS2 null mouse liver homogenates. Three mice were used per group. Very long-chain acyl-CoA synthetase is also known as fatty acid transporter protein 2. A complete list of all immunoprecipitated proteins is given in Table S1 with the 15 differentially coimmunoprecipitated proteins indicated.

a It was not possible to distinguish between Selenbp1/2 and Selenbp2 by LC-MS/MS.

**Figure 1:**
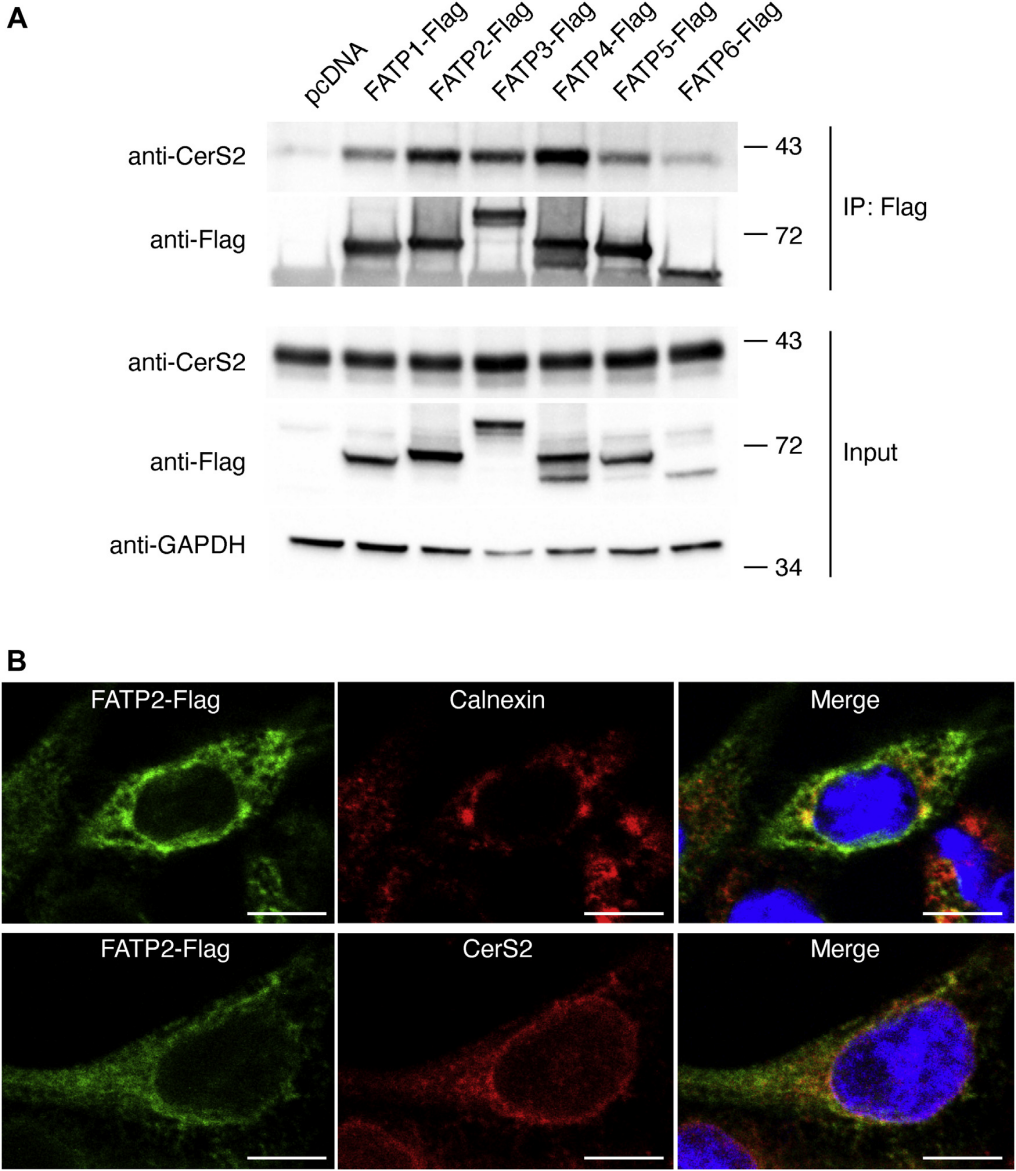
**CerS2 interacts with FATPs.***A*, Hek293T cells were transfected with all six FATPs, containing a C-terminal Flag tag, or with pcDNA. After 48 h, the cells were lysed and immunoprecipitated with an anti-Flag antibody. CerS2 was identified by Western blotting using a rabbit anti-CerS2 antibody (*upper panels*; IP-Flag). Levels of protein expression are shown in the *lower panels* (Input), and GADPH is shown as a loading control. The experiment was repeated three times with similar results. Mr markers (kDa) are indicated. *B*, Hek293T cells were transfected with FATP2-Flag for 48 h. The *upper panels* show that FATP2-Flag colocalizes with the ER marker, calnexin, and the *lower panels* show that FATP2-Flag colocalizes with CerS2. The experiment was repeated three times with similar results. Cell nuclei were labeled with the Hoechst stain. The bars represent 10 μm. CerS, ceramide synthase; ER, endoplasmic reticulum; FATP, fatty acid transport protein; HEK, human embryonic kidney.

The latter was directly tested by expressing all six members of the human FATP family in human embryonic kidney (Hek)293T cells followed by immunoprecipitation using an anti-CerS2 antibody. CerS2 coimmunoprecipitated with all six FATP members, with a higher level of coimmunoprecipitation with FATP2 and 4 (Fig. 1*A*), which may be related to the colocalization of FATP2 (Fig. 1*B*) and FATP4 (not shown) in the endoplasmic reticulum (ER).

**Figure 2:**
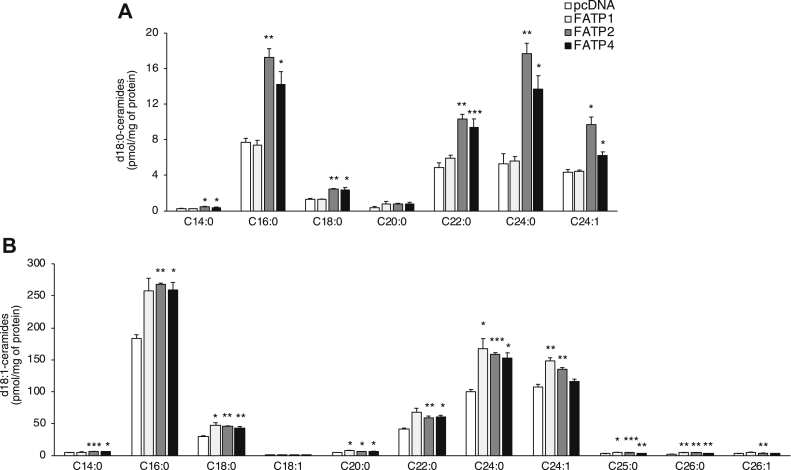
**Cellular dihydroceramide and ceramide levels are elevated upon transfection with FATPs.** Hek293T cells were transfected with FATP1, 2, or 4 for 48 h prior to analysis of (*A*) dihydroceramide (d18:0) and (*B*) ceramide (d18:1) levels by LC-MS/MS. The data are means ± S.E.M., n = 3. ∗*p* < 0.05; ∗∗*p* < 0.01; ∗∗∗*p* < 0.001. FATP, fatty acid transport protein; HEK, human embryonic kidney.

We next examined whether FATPs 1, 2, and 4 directly impact cellular SL levels. Hek293T cells were transfected with these three FATPs and SL levels measured 48 h later by liquid chromatography electrospray ionization tandem mass spectrometry. Levels of dihydroceramides (d18:0) were elevated upon transfection of FATP2 and FATP4 irrespective of the *N*-acyl chain length (Fig. 2*A*) (with the exception of C20-dihydroceramide). However, transfection with FATP1 had no effect on dihydroceramide levels. Ceramide levels were elevated upon transfection with all three FATPs, also irrespective of *N*-acyl chain length (Fig. 2*B*). No significant changes were detected in d18:1-hexosylceramides or d18:1-sphingomyelin levels, but a small decrease in d18:1/C24:0 and C24:1-lactosylceramide levels were observed (data not shown). Together, these data suggest that some FATPs, predominantly FATP2 and 4, can interact with CerS2 and enhance cellular dihydroceramide and ceramide levels, presumably by increasing the availability of acyl-CoAs.

### Catalytically active CerS and FATP are required for their interaction

**Figure 3:**
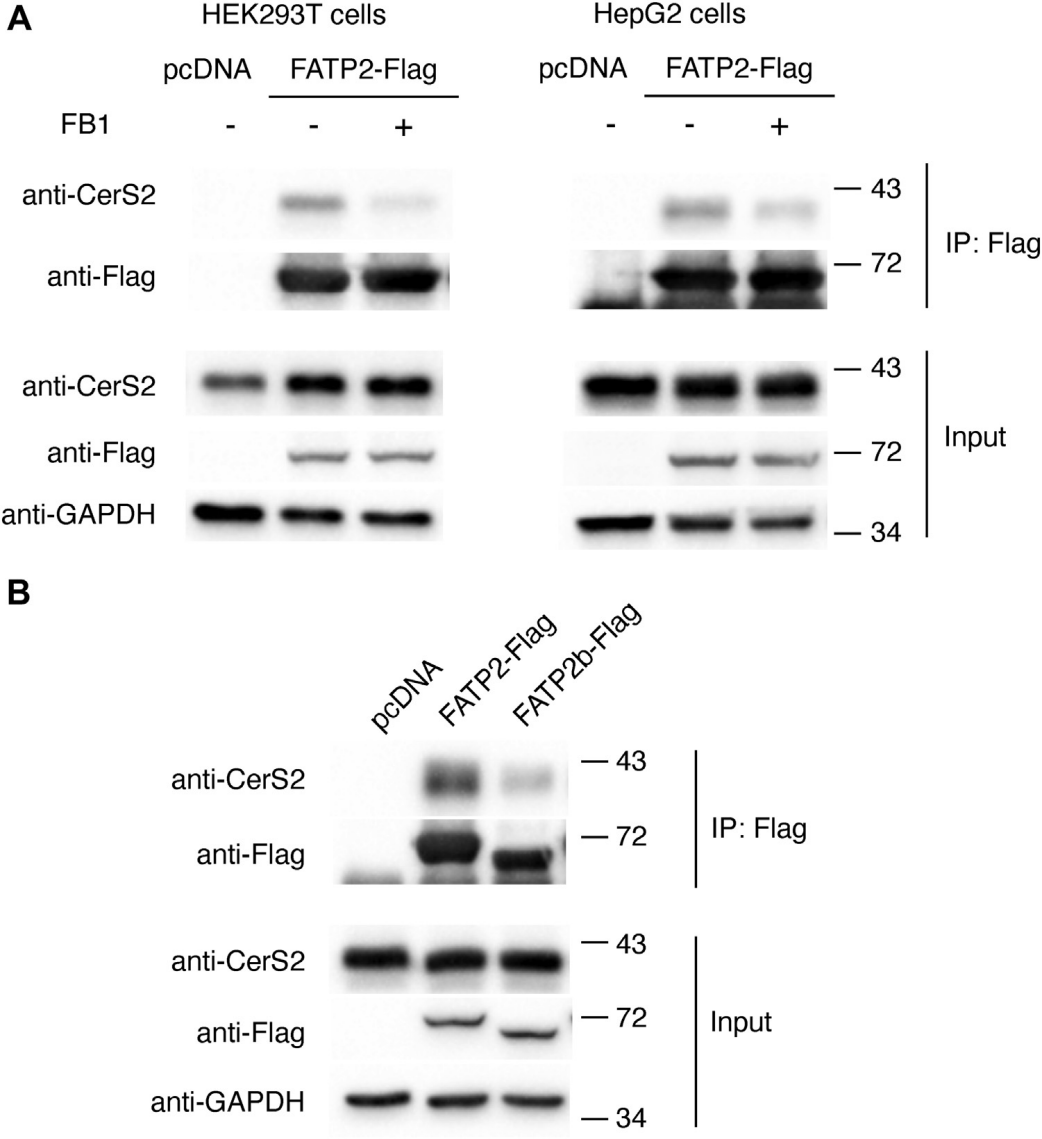
**Catalytically active CerS2 and FATP2 are required in order for them to interact.***A*, Hek293T and HepG2 cells were transfected with FATP2-Flag for 48 h. The cells were incubated with or without FB1 (20 μM) for 24 h prior to harvesting, lysis, and immunoprecipitation using an anti-Flag antibody. CerS2 was identified by Western blotting using an anti-CerS2 antibody (*upper panel*; IP-Flag). Levels of expression of CerS2 and FATP2-Flag are shown in the *lower panel* (Input), and GADPH is shown as a loading control. The experiment was repeated three times. Mr markers (kDa) are indicated. *B*, Hek293T cells were transfected with full-length FATP2 or with a splice variant (FATP2b) and immunoprecipitation performed as in *A*. The experiment was repeated three times with similar results. Mr markers (kDa) are indicated. CerS, ceramide synthase; FATP, fatty acid transport protein; FB1, fumonisin B1; HEK, human embryonic kidney.

Based on the assumption that FATPs interact with CerS in order to supply the acyl-CoAs needed for ceramide synthesis, we examined the extent of coimmunoprecipitation of the two proteins upon inhibition of their activity. First, Hek293T or HepG2 cells (which contain high levels of FATP2 (24)) were transfected with FATP2-Flag and treated with the CerS inhibitor, fumonisin B1 (FB1) (25). The extent of coimmunoprecipitation of CerS2 with FATP2 decreased in both cell types by ∼75% upon incubation with FB1 (Fig. 3*A*). Next, we compared the extent of coimmunoprecipitation using a splice variant of FATP2, FATP2b, which lacks exon 3 and is unable to synthesize VLC–acyl-CoAs even though it maintains its ability to import free fatty acids (26). This splice variant also showed significantly less binding (∼70%) to CerS2 (Fig. 3*B*). Together, these two results are consistent with the notion that the interaction of CerS2 and FATP2 depends on the maintenance of catalytic activity, either *via* the supply of VLC–acyl-CoAs and their utilization by CerS for the *N*-acylation of sphinganine, although we cannot exclude the possibility that FATP2 binds to CerS2 *via* the sequence encoded by exon 3.

### The C-terminus of CerS2 may regulate the interaction with FATP2

**Figure 4:**
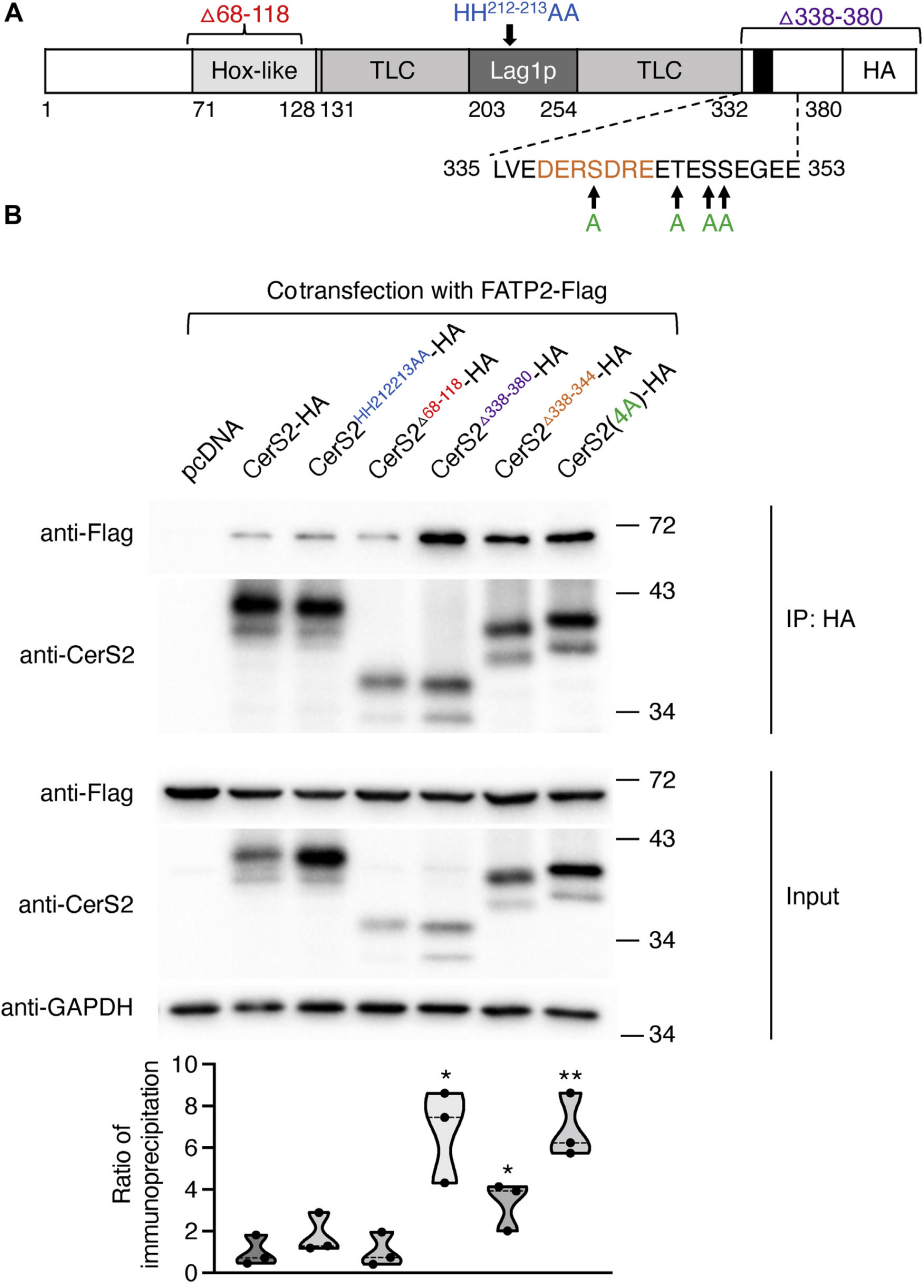
**Effect of CerS2 mutations on binding to FATP2.***A*, schematic representation of the domains of CerS2-HA with the site of the mutations (10, 27, 28) color coded as in *B*. The numbers indicate the position of amino acid residues in the sequence. *B*, CerS2^−/−^ Hek293T cells were transfected with pcDNA or CerS2-HA mutants along with FATP2-Flag. After 48 h, the cells were lysed and immunoprecipitated with an anti-HA antibody. FATP2-Flag was identified using an anti-Flag antibody (*upper panel*; IP-HA). Levels of protein expression are shown in the *middle panels* (input). GAPDH is shown as a loading control. Mr markers (kDa) are indicated. The experiment was repeated three times with similar results. The *lower panel* shows quantification of three independent coimmunoprecipitation experiments. The data are shown as a truncated violin plot with individual values. The Student *t* test was used to compare each group to CerS2-HA. ∗*p* < 0.05; ∗∗*p* < 0.01. CerS, ceramide synthase; FATP, fatty acid transport protein; HEK, human embryonic kidney.

To attempt to determine which structural domains of CerS2 are involved in the binding to FATP2, we took advantage of a series of CerS2 mutations and deletion constructs (10, 27, 28). Deletion of the Hox-like domain (27) had no effect on the extent of immunoprecipitation of CerS2 with FATP2 in CerS2^−/−^ HEK cells and neither did replacement of the two putative active site residues (HH^212–213^AA) (28) (Fig. 4). The reason that incubation with FB1 decreases the extent of immunoprecipitation between CerS2 and FATP2, whereas deletion of the putative active site residues does not, is currently unknown. However, it should be noted that FB1 inhibits CerS by competition with sphinganine (due to their structural similarities), perhaps inducing a structural, kinetic, or allosteric change which does not occur upon deletion of the putative active site histidine residues (29).

Unexpectedly, deletion of the C-terminus, deletion of a novel heptapeptide found uniquely in the C-terminus of CerS (30), or replacement of four putative phosphorylation sites (10) in the C-terminus increased the extent of coimmunoprecipitation (Fig. 4). The latter is of particular interest since phosphorylation of these four residues has been implicated in the regulation of *de novo* ceramide synthesis (10, 31, 32), and our data imply that phosphorylation may actually regulate the mode of interaction of CerS with FATP2, which could directly impinge upon CerS activity. Alternatively, the C-terminus of CerS appears to be unstructured in most structural prediction programs, and it may be that mutating or deleting this region nonspecifically enhances the accessibility of the CerS to interact with FATP. However, none of the five mutations or deletion constructs reduced the extent of coimmunoprecipitation with CerS2, such that further work is required to unambiguously determine the precise structural features by which CerS and FATPs interact.

### An FATP2 inhibitor down-regulates *de novo* dihydroceramide synthesis

**Figure 5:**
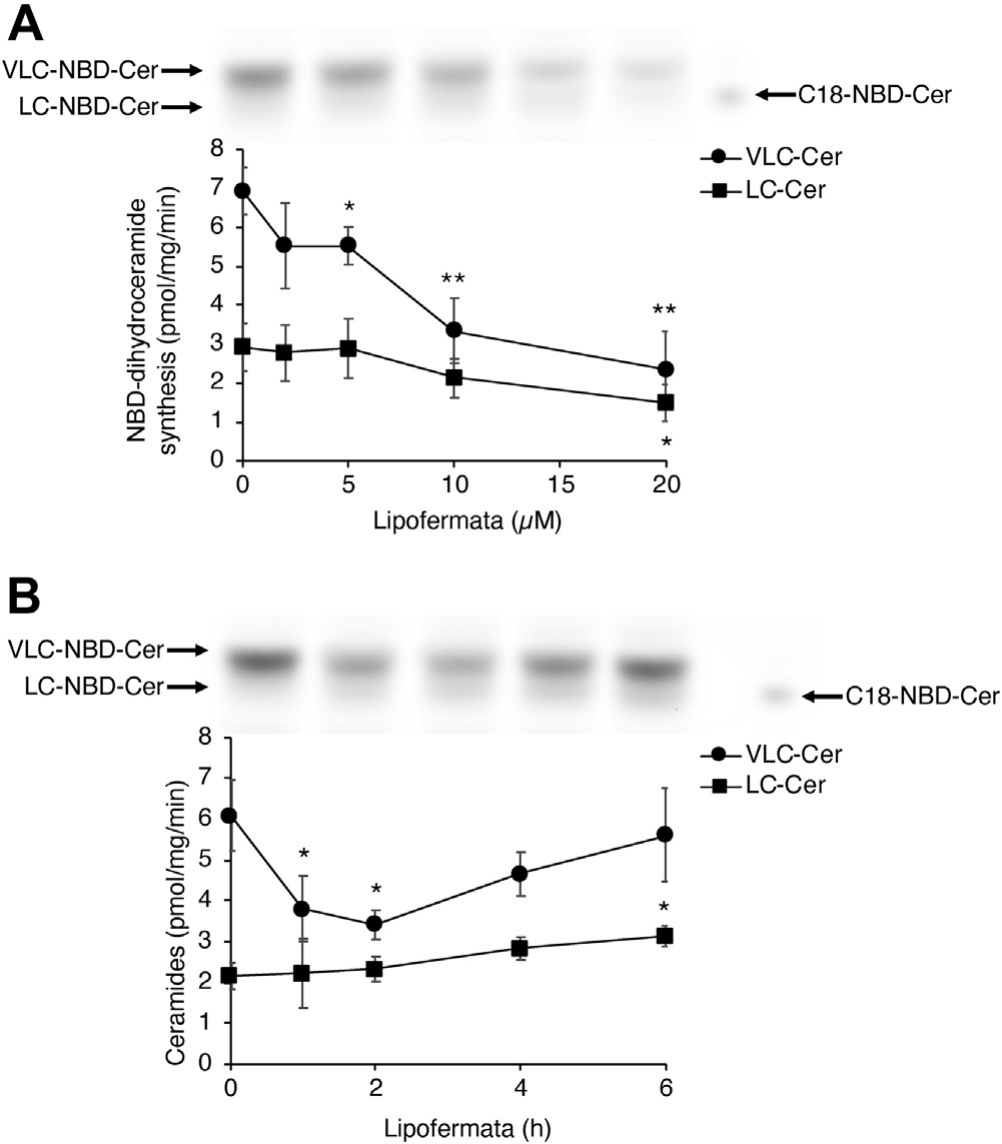
**Lipofermata inhibits *de novo* dihydroceramide synthesis.** HepG2 cells were cultured for 4 days and were then incubated with (*A*) increasing concentrations of lipofermata for 1 h or with (*B*) 10 μM lipofermata for increasing times. The cells were incubated with NBD-Sph for the last 25 min of the incubation period with lipofermata. LC- (*squares*) and VLC–NBD-ceramides (*circles*) were separated by TLC, examples of which are given in each panel. The data are means ± S.D., n = 3. The Student *t* test was used to compare each group to untreated controls ∗*p* < 0.05; ∗∗*p* < 0.01. LC, long-chain; NBD-Sph, NBD-Sphinganine; VLC, very long-chain.

Lipofermata is an inhibitor of FATP2 (33) and is believed to directly impact tumor cell growth *via* inhibition of FATP2 and to also prevent lipotoxicity induced by fatty acids (21, 22, 34, 35). We examined whether the inhibition of FATP2 by lipofermata affects the rate of dihydroceramide synthesis in HepG2 cells by metabolic labeling using NBD-Sphinganine, which is converted to NBD-dihydroceramide. Levels of both VLC- and LC-NBD-dihydroceramide synthesis decreased in a concentration-dependent manner (Fig. 5*A*) upon incubation with lipofermata. The levels of VLC-NBD-dihydroceramide synthesis also decreased in a time-dependent manner (Fig. 5*B*), but reverted to control levels after 4 h incubation, suggesting that the efficacy of lipofermata decreases after longer times of incubation. This data implies that any potential therapeutic effects of lipofermata may not only be related to its ability to inhibit FATP2, but may also be affected by the levels of other lipids whose levels can be modulated by FATP2, among them (dihydro)ceramide, a critical lipid involved in regulating normal cell and tumor cell growth (36).

## Discussion

In the current study, we extend the number of proteins that have been shown to interact with the CerSs by demonstrating that the six isoforms of FATP interact with CerS2 to some extent. While this interaction appears to be generic, suggesting that common structural features determine the mode of interaction, the specific interaction between CerS2 and FATP2 appears most relevant since both proteins are located in the ER (6, 37), and both are related to either the generation or utilization of VLC–acyl-CoAs. This is consistent with lipidomics analysis in which FATP2 (and FATP4) directly impacted the *de novo* SL synthesis pathway (note that FATP4 is also located in the ER), whereas FATP1 did not impact *de novo* synthesis but did affect SLs associated with the recycling pathway, consistent with the suggested subcellular localization of FATP1 in the plasma membrane (38, 39), perhaps suggesting that FATP family members need to be adjacent to the CerS to allow their interaction in cells. Thus, although CerS2 may be able to interact with all FATPs upon coimmunoprecipitation, the interaction with FATP2 may be of most relevance in cell physiology.

**Figure 6:**
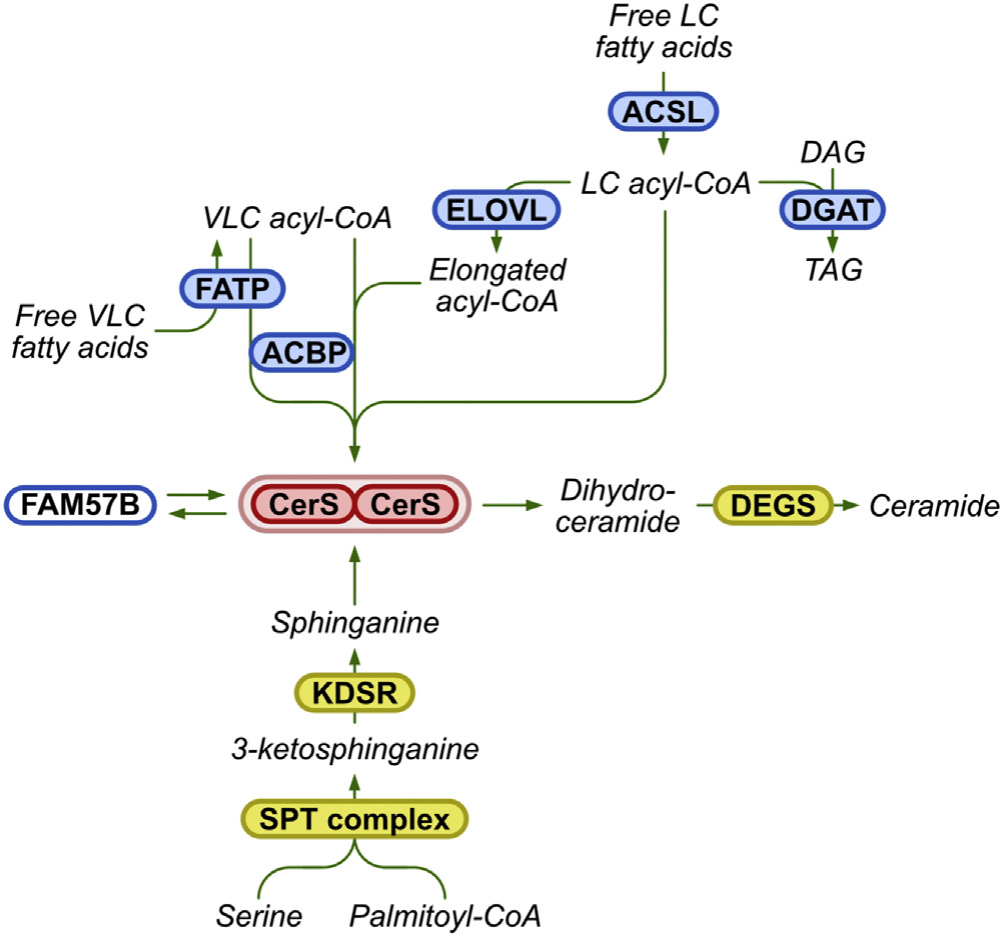
**A scheme of CerS-interacting proteins.** The *upper branch* shows the proteins involved in the pathway by which acyl-CoAs are supplied to the CerS, with enzymes encircled in *blue* and metabolic intermediates in *black*. The *lower branch* shows the enzymes (encircled in *yellow*) involved in ceramide synthesis. CerS are shown as a dimer (9), with a related protein, FAM57B, indicated due to its ability to regulate CerS activity and levels (42). For further details, see text. ACBP, acyl-CoA-binding protein; ACSL, long-chain acyl-CoA synthetase; CerS, ceramide synthase; DAG, diacylglycerol; DEGS, dihydroceramide desaturase; ELOVL, elongation of very long-chain fatty acids protein; FATP, fatty acid transport protein; KDSR, 3-ketosphinganine reductase; LC, long-chain; SPT, serine palmitoyltransferase; TAG, triacylglycerol; VLC, very long-chain.

FATP joins the growing pantheon of proteins that intersect with the metabolic pathway of ceramide synthesis (Fig. 6). While the generation of sphinganine occurs in the membrane of the ER *via* the sequential action of two ER-embedded proteins, the generation of acyl-CoA occurs in a wider variety of subcellular locations. Thus, free fatty acids can be taken-up by cells at the plasma membrane *via* a variety of transporters (40), including some FATPs, prior to the generation of fatty acyl-CoAs. The VLC–acyl-CoAs generated by FATP2 are then used for VLC-ceramide generation, *via* a pathway that also includes acyl-CoA–binding protein (12). Another pathway of generation of acyl-CoAs is *via* the action of LC-acyl-CoA synthetases, which also bind to CerS (13), and can provide acyl-CoA precursors for the elongation steps mediated *via* ELOVLs (11). Thus, two distinct branches become apparent upon analysis of the supply of substrates for the CerSs (Fig. 6), implying multiple finely tuned mechanisms (41) involved in the intricate regulation of the rate of (dihydro)ceramide synthesis. In addition, CerS activity can also be regulated by dimer formation, which depends on a short C-terminal motif (30), and by interaction with another member of the same protein family, FAM57B (42). While many other metabolic pathways are also tightly regulated, the critical roles that ceramide plays as a precursor to a whole gamete of SLs (43), and as a cellular signaling molecule, apparently necessitates these remarkably complex mechanisms of regulation.

One of the signaling pathways with which ceramide is associated is cell death, with ceramide generally considered to be proapoptotic (44, 45), although the roles of dihydroceramide in cell death pathways are less well established (46). Interestingly, incubation of cells with the FATP inhibitor, lipofermata, reduced levels of dihydroceramide synthesis. Lipofermata has been proposed as an anticancer agent (21, 22) *via* its interaction with FATP, although no studies are available prior to the current one examining whether lipofermata also impacts the ceramide pathway. Our data now show that this is the case and strengthen the notion that manipulating the pathway of fatty acyl-CoA generation is likely to impact a wide variety of down-stream lipids, not least the SLs, which utilize two acyl-CoA moieties in the initial steps of their synthesis.

## Experimental procedures

### Materials

NBD-Sphinganine and C18:0-NBD-ceramide were from Avanti Polar Lipids. Defatted-bovine serum albumin, FB1, a protease inhibitor cocktail, rabbit anti-CerS2, mouse anti-Flag, and mouse anti-GAPDH antibodies were from Sigma. Lipofermata was from Cayman Chemicals. A mouse anti-CerS2 antibody was from Santa Cruz and a rabbit anti-FATP2 antibody was from Proteintech. A rabbit anti-Calnexin antibody was purchased from Abcam. Horseradish peroxidase was from the Jackson Laboratory. An enhanced chemiluminescence detection system was from Thermo Scientific. 4x Laemmli sample buffer was from Bio-rad. Silica gel 60 TLC plates were from Merck. All the solvents were of analytical grade and were purchased from Bio-Lab. Anti-DYKDDDDK G1 Affinity Resin, pcDNA 3.1(+) vectors containing FATP1-6, and FATP2b with a C-terminus Flag-tag were from GenScript.

### Animals

Livers from WT and CerS2 null mice (23) were perfused with cold PBS. All mouse studies were approved by the Weizmann Institute of Science Animal Care Committee according to National Institutes of Health's Guidelines for Animal Care.

### Proteomics

Proteomics was performed essentially as described (47); see also Supplemental Methods. Briefly, protein A agarose beads with CerS2 were incubated with 5% sodium dodecyl sulfate in 50 mM Tris–HCl and loaded onto S-Trap microcolumns (ProtiFi). Eluted samples after digestion with trypsin (1:50, trypsin/protein, wt/wt) were loaded onto a split-less nano ultra performance liquid chromatography column (10 kpsi nanoAcquity; Waters). The nano ultra performance liquid chromatography was coupled online through a nano-electrospray ionization emitter (10 μm tip; New Objective) to a quadrupole orbitrap mass spectrometer (Q Exactive Plus, Thermo Scientific) using a FlexIon nanospray apparatus (Proxeon). Raw data was processed with MaxQuant v1.6.0.16. The data was analyzed with the Andromeda search engine against the Uniprot mouse proteome database (48). Label-free quantification intensities were calculated and used for further calculation by Perseus v1.6.0.7. Decoy hits were filtered out as well as proteins that were identified on the basis of only one modified peptide. The data was further filtered to include only proteins with at least two valid values in at least one of the groups. A Student’s *t* test, after logarithmic transformation, was used to identify significant differences across biological replicates. Fold-change was calculated based on the ratio of geometric means of the different groups.

### Liquid chromatography electrospray ionization tandem mass spectrometry

Sphingolipid analysis by liquid chromatography electrospray ionization tandem mass spectrometry using an ABI 4000 quadrupole-linear ion trap mass spectrometer was performed as described (30, 49).

### CerS constructs

CerS constructs (Fig. 4) were subcloned using restriction-free cloning (50) from CerS2 in a pcDNA 3.1(+) vector containing a C-terminus HA tag. Primers are given in Table 2. All sequences were confirmed prior to use.

**Table 2:** Primers used for subcloning

Construct	Primers	
CerS2^H212A-H213A^-HA	F	CAAGGAACAGATCATCGCCGCTGTGGCCACCATCATTC
	R	GAATGATGGTGGCCACAGCGGCGATGATCTGTTCCTTG
CerS2^Δ68–118^-HA	F	CCACTGGCTGCCCTCCGTCGCCGCCGCAAC
	R	GTTGCGGCGGCGACGGAGGGCAGCCAGTGG
CerS2^Δ335–380^-HA	F	GGCCCACAAGTTCATAACTGGAAAGTACCCATACGATGTTCCAGATTAC
	R	GTAATCTGGAACATCGTATGGGTACTTTCCAGTTATGAACTTGTGGGCC
CerS2^Δ338–344^-HA	F	CAAGTTCATAACTGGAAAGCTGGTAGAAGAAACAGAGAGCTCAGAGGGGGAG
	R	CTCCCCCTCTGAGCTCTCTGTTTCTTCTACCAGCTTTCCAGTTATGAACTTG
CerS2(4A)-HA	F	GCAGACCGGGAAGAAGCAGAGGCAGCAGAGGGGGAGGAGG
	R	CCTCCTCCCCCTCTGCTGCCTCTGCTTCTTCCCGGTCTGC

Abbreviations: F, forward; R, reverse.

### Cell culture and transfection

Human embryonic kidney 293T cells and CerS2^−/−^ Hek293T cells (51) were cultured in Dulbecco’s modified Eagle’s medium (DMEM) supplemented with 10% fetal bovine serum, 100 IU/ml penicillin, 100 μg/ml streptomycin, and 110 μg/ml sodium pyruvate. Transfections were performed with the polyethylenimine reagent using 8 μg of plasmid per 10 cm culture dish. The cells were removed from culture dishes after 48 h and washed twice with PBS on ice before immunoprecipitation. HepG2 hepatoma cells were cultured in DMEM supplemented as above but including Glutamax (Thermo Fisher Scientific). The cells were maintained at 37 °C in a humidified atmosphere containing 5% CO_2_.

### Coimmunoprecipitation

Mouse liver was homogenized in lysis buffer (150 mM NaCl, 20 mM Hepes pH 7.5) containing 1% digitonin (v/v) and a protease inhibitor cocktail. After centrifugation (15,000*g*_av_, 10 min, 4 °C), protein was determined in the supernatant using the bicinchoninic acid reagent (Cyanagen). One microgram of a rabbit anti-CerS2 antibody was used to pull-down CerS2 from liver lysates (2 mg protein). After overnight incubation, protein A agarose beads were added and gently rotated (4 °C, 2 h). The beads were subsequently washed four times with 1 ml of lysis buffer.

Cultured cells were transfected for 48 h and then washed twice with ice-cold PBS prior to lysis in 150 mM NaCl, 50 mM Tris–HCl pH 7.4, 1% Nonidet P-40 containing a protease inhibitor cocktail. Cell homogenates were centrifuged (15,000*g*_av_, 4 °C, 10 min), and the supernatant was incubated overnight with an anti-DYKDDDDK G1 Affinity Resin or a rabbit anti-HA antibody in a tube rotator. Following incubation with a rabbit anti-HA antibody, protein A agarose beads (Santa Cruz) were added and gently rotated for 2 h. Beads were collected and washed three times with 1 ml of lysis buffer. All procedures were performed at 4 °C on ice. The beads were heated (80 °C, 10 min) with sample buffer to elute Flag-bound proteins. The eluates were stored at −20 °C.

### Western blotting

Proteins were separated by 10% SDS-PAGE and transferred to nitrocellulose membranes. Flag-tagged constructs were identified using a mouse anti-Flag antibody (1:5000 dilution) and goat anti-mouse horseradish peroxidase (1:10,000 dilution) as the secondary antibody. CerS2 proteins were detected using a rabbit anti-CerS2 antibody or a mouse anti-CerS2 antibody followed by incubation with a goat anti-rabbit or antimouse horseradish peroxidase antibody (1:10,000 dilution). Equal loading was confirmed using a mouse anti-GAPDH antibody (1:5000 dilution). Detection was performed using the enhanced chemiluminescence detection system. Protein bands were imaged and quantified using the ChemiDoc MP imaging system (Bio-rad).

### Immunocytochemistry

Human embryonic kidney 293T cells were fixed with methanol:acetone (1:1, v/v) for 7 min on ice followed by incubation with 5% donkey serum/1% bovine serum albumin (v/v) in PBS for 1 h. Cells were then incubated with a rabbit anti-calnexin antibody (1:300 dilution) or with a mouse anti-CerS2 antibody (1:100 dilution) along with a mouse anti-Flag antibody (1:1000 dilution) or a rabbit anti-FATP2 antibody (1:50 dilution) at 4 °C overnight. After three washes with PBS, the cells were incubated with Alexa-488 conjugated donkey anti-mouse and Alexa-647 dye-conjugated donkey anti-rabbit secondary antibodies (1:1000 dilution) in blocking buffer for 1 h. Hoechst 33342 (Invitrogen) was used to label nuclei, and the cells were examined by confocal laser scanning microscopy using an Olympus FluoView FV500 imaging system.

### Metabolic labeling

Cells were incubated in serum-free medium for 24 h prior to incubation with 20 μM lipofermata (dissolved in 0.1% DMSO) in serum-free medium. After various times of incubation with lipofermata, 3 μM NBD-Sphinganine was added for 25 min. After washing the cells with PBS, cells were homogenized in ice-cold distilled water and an aliquot taken for protein determination. The remaining homogenate was lysed in chloroform/methanol (1:2, v/v) to extract lipids (52). Lipids were dried under N_2_, resuspended in chloroform/methanol (9:1, v/v), and separated by TLC using chloroform/methanol/2 M NH_4_OH (40:10:1, v/v/v) as the developing solvent. NBD-labeled LC- and VLC–NBD-ceramides were visualized using an Amersham Typhoon 5 biomolecular fluorescence imager and quantified by ImageQuantTL software (GE Healthcare). C18:0-NBD-ceramide was used as a marker.

### Statistics

Statistical significance was assessed using an unpaired two-tailed Student’s t test. ∗*p* < 0.05; ∗∗*p* < 0.01; ∗∗∗*p* < 0.001.

## Data availability

The mass spectrometry proteomics data was deposited in the ProteomeXchange Consortium *via* the PRIDE (53) partner repository with the dataset identifier PXD031088.

## Supporting information

This article contains supporting information (47).

## Conflict of interest

The authors declare that they have no conflicts of interest.
