# Supplementary material for: Fatty acid transport protein 2 interacts with ceramide synthase 2 to promote ceramide synthesis
Source: J Biol Chem. 2022 Feb 16;298(4):101735. doi: 10.1016/j.jbc.2022.101735 (PMC8931434; doi:10.1016/j.jbc.2022.101735)
Supplement: Supplemental Methods [file mmc1.docx]

**Supplemental Methods**

**Sample preparation**

Eluted proteins from 3 biological replicates per group were reduced with 5 mM dithiothreitol and alkylated with 10 mM iodoacetamide in the dark. Samples were loaded onto S-Trap microcolumns (Protifi, USA) according to the manufacturer’s instructions. In brief, after loading, samples were washed with 90:10% methanol/50 mM ammonium bicarbonate. Samples were then digested with 500 ng trypsin for 1.5 h at 47°C. The digested peptides were eluted using 50 mM ammonium bicarbonate; trypsin was added to this fraction and incubated overnight at 37°C. Two more elutions were performed using 0.2% formic acid and 0.2% formic acid in 50% acetonitrile (v/v). The three elutions were pooled together and vacuum-centrifuged to dry. Samples were stored at −80°C.

**Liquid chromatography**

ULC/MS grade solvents were used for chromatography. Samples were loaded using split-less nano-Ultra Performance Liquid Chromatography (10 kpsi M-Class; Waters, Milford, MA, USA). The mobile phase was: A) H2O + 0.1% formic acid and B) acetonitrile + 0.1% formic acid. Desalting of the samples was performed online using a reversed-phase Symmetry C18 trapping column (180 µm internal diameter, 20 mm length, 5 µm particle size; Waters). Peptides were separated using a T3 HSS nano-column (75 µm internal diameter, 250 mm length, 1.8 µm particle size; Waters) at 0.35 µL/min. Peptides were eluted from the column into the mass spectrometer using the following gradient: 4% to 30%B in 50 min, 30% to 90%B in 5 min, maintained at 90% for 5 min and then back to initial conditions.

**Mass Spectrometry**

The nanoUPLC was coupled online through a nanoESI emitter (10 μm tip; New Objective; Woburn, MA, USA) to a quadrupole orbitrap mass spectrometer (Q Exactive Plus, Thermo Scientific) using a FlexIon nanospray apparatus (Proxeon). Data was acquired in data dependent acquisition (DDA) mode using a Top10 method. MS1 resolution was set to 70,000 (at 400 m/z), mass range of 375-1650 m/z, AGC of 3e6 and maximum injection time was set to 60 msec. MS2 resolution was set to 17,500, quadrupole isolation 1.7 m/z, AGC of 1e6, dynamic exclusion of 30 sec and maximum injection time of 60 msec.

**Data processing**

Raw data was processed with MaxQuant v1.6.0.16. The data was searched with the Andromeda search engine against the Uniprot mouse proteome database (16,962 entries, Jan 5^th^ 2018 release version) appended with common laboratory protein contaminants and the following modifications: carbamidomethylation of C as a fixed modification, oxidation of M and deamidation of N and Q as variables. The enzyme used was Trypsin (fully specific), maximal 2 mis-cleavages allowed, precursor mass tolerance was set to 10 ppm, while the fragment mass tolerance was kept at 20 ppm. The maximal FDR was set to 0.01 using a reverse decoy database strategy. The threshold score for the Q value was kept at 0.01. LFQ intensities (Label-Free Quantification) were calculated and used for further calculations using Perseus v1.6.0.7. Decoy hits were filtered out, as well as proteins that were identified on the basis of one modified peptide only. The data was further filtered to include only proteins with at least 2 valid values in at least one of the groups and then the data was imputed and GO annotations added. A Student’s t-Test, after logarithmic transformation, was used to identify significant differences across the biological replica. Fold-changes were calculated based on the ratio of geometric means of the different compared groups.
